# Different types of screen-based sedentary time and anxiety in adolescents: Video games may be more important

**DOI:** 10.3389/fpubh.2022.918234

**Published:** 2022-09-06

**Authors:** Sitong Chen, Cain C. T. Clark, Zhanbing Ren

**Affiliations:** ^1^School of Physical Education and Sport, Shenzhen University, Shenzhen, China; ^2^Faculty of Health and Life Sciences, Coventry University, Coventry, United Kingdom

**Keywords:** screen time, types, anxiety, cross-sectional survey, adolescent

## Abstract

**Aim:**

Evidence demonstrates the negative impact of excessive screen-based sedentary time (screen-based sedentary behavior; SSB) on mental health in adolescents. However, little is known regarding the associations between different types of SSBs and anxiety symptoms in adolescents. Thus, this study sought to explore the associations between different types of SSBs and anxiety symptoms in a sample of Chinese adolescents.

**Methods:**

A web-based questionnaire survey was used to collect data. In total, 1,998 study participants conveniently recruited in Guangdong Province completed the questionnaire. Of them, 1,331 study participants provided valid data for variables of interest. SSB was categorized into television/movie time, video game time, and internet-surfing time. Anxiety symptoms were assessed using *Zung* Self-Rating Anxiety Scale, a validated assessment in Chinese youth populations. Generalized linear models were used to explore the associations between different types of SSBs and anxiety symptoms.

**Results:**

In adolescents, video game time of 6 or more h was positively and significantly associated with anxiety symptoms (odds ratio = 5.25, 95% CI: 1.86–14.84, *p* < 0.01). This association was also observed specifically in boys (odds ratio = 5.12, 95% CI: 1.56–17.44, *p* < 0.05); however, in girls, there were no associations between different types of SSBs and anxiety symptoms.

**Conclusion:**

Interventions aiming at reducing video games in adolescents, especially in boys, should be designed to prevent anxiety symptoms. This kind of intervention should also take sex differences into consideration. Future studies are encouraged to confirm the veracity of the findings in this study.

## Introduction

Anxiety is one of the most common mental health problems in adolescents worldwide (1). Anxiety symptoms include both psychiatric (e.g., worries, uneasiness, and phobia) and somatic symptoms (e.g., shortness of breath, chest distress, and muscle tension) (2). Many studies have shown that adolescent anxiety is associated with a series of adverse outcomes, such as negative attention bias, substance abuse (e.g., nicotine and narcotics), sleep (SLP) disturbance, and even suicide (3–5). Although numerous studies pertaining to anxiety have been conducted in adult samples, research on adolescents, especially in the Chinese population, is sparse. Currently, according to the latest report released by the National Health Commission of China, the prevalence of anxiety in Chinese adolescents indicates an upward trend, therein highlighting the importance of further examining anxiety for prevention and intervention programs. The prevalence of anxiety in adolescents has been found to vary considerably across regions and populations. For instance, a cross-sectional study consisting of Indian adolescents aged 10–19 years found that 16.6% of the participants had anxiety disorders (6). According to the data from the 2016 National Survey of Children's Health in the United States, 10.5% of adolescents were diagnosed with anxiety problems (7). Moreover, a recent study showed the prevalence of anxiety was 11.8% among Spanish adolescents (8). In addition, a report on the 12-month prevalence of anxiety in Hong Kong showed a prevalence of 5.1% (9). With respect to mainland China, a study covering 5,249 students showed that the prevalence of anxiety was 14.1% (10).

Screen-based sedentary behavior (SSB) is an important correlate of anxiety in adolescents (11). Specifically, evidence suggests that the association between television (TV) viewing and anxiety is not significant in adolescents (11, 12), but only video gaming is positively associated with anxiety in Canadian adolescents. Further, the prevalence of anxiety was reported to have increased by 6.8%, in comparison with adolescents who spent <2 h of SSB per day (13). In addition, one study revealed that passive SSB (such as watching TV and video), but not active SSB (such as chatting, video gaming, working, and surfing the internet), is associated with anxiety, and adolescents who spent 4 or more h engaged in passive SSB were more likely to be diagnosed as anxiety, in comparison with those reporting <2 h per day (14). Nevertheless, due to a large heterogeneity across different studies, the association between SSB and anxiety in adolescents is currently unclear. Moreover, across the published literature, there are several other limitations that should be noted, in particular, SSB typology. Indeed, there is a dearth of research focusing on specific types of SSBs and their roles on mental health. This gap represents a distinct barrier to better understanding the associations between SSB and anxiety, as well as other mental health indicators in adolescents. Another gap is that most of the previous studies were based on western populations, while little evidence comes from Chinese populations. A final research gap is that previous studies have tended to omit some important control variables related to anxiety symptoms, such as SLP duration.

Therefore, it is necessary for research to specifically address the abovementioned research gaps to provide more compelling evidence. Accordingly, this study sought to explore the associations between different types of SSBs and anxiety symptoms in a sample of Chinese adolescents (situated in Guangdong Province).

## Methods

### Study design and participants

This cross-sectional study was designed to assess adolescents' physical and mental health. We randomly selected one middle school (there are three grades in middle school according to China's education system) in each city (11 cities in our study) in the Guangdong Province. In each grade, students from three classes were randomly selected. A total of 3,465 students were invited. Of them, 1,998 students and their parents/guardians completed the survey (response rate = 57.7%). Of the 1,998 study participants, 1,331 study participants provided valid data pertaining to the variables of interest in this study. Prior to data collection, the headmaster of each school, study participants, and their parents/guardians were informed of study aims and instructions. Each study participant provided written consent to take part in this study. To protect study participants' privacy, data were collected and analyzed anonymously. This study was approved by the Institutional Review Board at Shenzhen University.

### Measures

#### Sociodemographic

Study participants were asked to self-report the following information, such as sex (boy or girl), grade (7, 8, or 9), ethnicity (Han or minority), residence (urban or rural), height (cm), weight (kg), perceived family affluence (scores from 0 to 10; using Family Affluence Scale), and siblings (none, one, two, three, four, or more).

#### Study outcome (anxiety symptoms)

Anxiety symptoms were assessed using a 20-item self-report questionnaire, the *Zung* Self-Rating Anxiety Scale (Z-SAS). The Z-SAS includes measures of state and trait anxiety based on scoring in four groups of manifestations: cognitive, autonomic, motor, and central nervous system symptoms. Responses to each item range from 1 (a little of the time) to 4 (most of the time) with higher scores indicating more severe anxiety. Standard scores were used in the statistical analysis of our study. We grouped study participants into tested as anxiety or not according to the cutoff point (40 scores) mentioned within the Z-SAS (15), and this categorization was used in the further statistical analysis as an outcome.

#### Study exposure (screen-based sedentary time, SSB)

Screen-based sedentary time was assessed by the Health Behavior in School-aged Children (HBSC) questionnaire (16), the questions included: (1) How many hours did you spend watching TV or movies in your leisure time on weekdays and weekend days over the past week, respectively? (2) How many hours did you spend playing video games in your leisure time on weekdays and weekend days over the past week, respectively? (3) How many hours did you spend in activities using electronic screen-based devices for internet-surfing in leisure time on weekdays and weekend days over the past week, respectively? The responses to these questions were 0.5–7 hours (or more). The average daily SSB hours were calculated using the following formula: average daily screen time (ST) hours = (ST hours on weekdays × 5 + ST hours on weekend × 2)/7. In the context of this study, ST was categorized into TV/movie time, video game time, and internet-surfing time. According to the measurement, in our study, different types of SSBs consisted of TV/movie time, video game time, and internet-surfing time. Then, we grouped different types of SSBs into the following categories: 0–2, 2–4, 4–6, and 6 or more hours per day.

#### Other behavioral control variables

Sleep duration was measured by the items derived from the HBSC questionnaire (16): (1) When do you usually go to bed if you have to go to school in the next morning? (2) When do you usually go to bed at weekends or during holidays? (3) When do you usually wake up on school mornings? (4) When do you usually wake up at weekends? The answers to items 1–4 were as follows: (1) no later than 21:00; 21:30; 22:00; 22:30; 23:00; 23:30; 24:00; 00:30; 01:00; 01:30; 02:00, or later; (2) no later than 21:00; 21:30; 22:00; 22:30; 23:00; 23:30; 24:00; 00:30; 01:00; 01:30; 02:00; 02:30; 03:00; 03:30; 04:00, or later; (3) no later than 05:00; 05:30; 06:00; 06:30; 07:00; 07:30; 08:00, or later; and (4) no later than 07:00; 07:30; 08:00; 08:30; 09:00; 09:30; 10:00; 10:30; 11:00; 11:30; 12:00; 12:30; 13:00; 13:30; 14:00, or later. Participants' response was used to calculate the SLP duration of the night on weekdays and the weekend, respectively. Then, the average SLP duration per night (hours) was calculated using the following formula: average SLP duration = (SLP duration per night on weekdays × 5 + SLP duration per night on weekends × 2)/7. In the statistical analysis, the variable of SLP was treated as continuous.

Moderate-to-vigorous physical activity (MVPA) was assessed by the HBSC (16), i.e., questionnaire that has been used in numerous Chinese epidemiological surveys (17–19). One question pertains to how many days for engagement in physical activity that increases your heartbeat and makes you breathe hard some of the times (such as physical education time, exercise, sports training, and various regular daily activities, such as brisk walking, hiking, and excursion) (e.g., physical education, exercise, and sports participations). The responses were 0–7 days. In the statistical analysis, the variable of MVPA was treated as continuous.

### Statistical analysis

All statistical analyses were conducted using SPSS version 25.0. Descriptive statistics was used to report the frequency and percentage (%) of categorical variables (e.g., sex and residence) and the mean and standard deviation (SD) of continuous variables (body mass index; BMI). The chi-square test was used to examine the sex differences across all categorical variables and the Student's *t*-test was used to examine the sex difference across all the continuous variables. As the study outcome was a binary variable, logistic regression within Generalized Linear Models (GLMs) was used to assess the associations between different types of SSBs and anxiety. Within GLMs, Maximum Likelihood Estimation (MLE) and Robust Estimator were used to examine the associations between different types of SSBs and anxiety. Adjusted odds ratios with 95% confidence intervals (95% CIs) were reported, and statistical significance was defined, *a priori*, as *p* < 0.05 (two-sided).

## Results

Table 1 summarizes the study sample characteristics. In total, 1,331 adolescents (grades 7–9) were included in the final analysis. In overall sample, 51.31% were boys (*n* = 683) while 48.69% were girls (*n* = 648). Most of the samples were from Chinese Han ethnicity (96.69%) and from urban areas (72.43%). In total, 84.3% of samples reported that they have one or more siblings, and the mean BMI in the samples was 18.99 kg/m^2^ (± 4.08). Overall, the study samples' accumulated days for MVPA was 5.05 (± 2.08), with a statistically significant difference between the sexes (boys: 5.43 vs. girls: 4.65, *p* < 0.001). The mean of SLP was 8.42 h (± 1.29) and there was no significant sex difference (*p* = 0.076). For three different types of SSBs, namely, TV/movie time, video game time, and internet-surfing time, their mean times were 2.29 (± 1.40), 2.41 (± 1.44), and 3.27 (± 1.51) h per day, respectively. The mean score of anxiety (assessed by the *Zung* scale) was 40.23 (± 8.71), with a statistically significant sex difference (girls [40.82 ± 9.22] > boys [39.66 ± 8.16], *p* < 0.005). More details are reported in Table 1.

**Table 1:** Sample characteristics of this study.

		**Total**		**Male**		**Female**		***p***
		***n***	***%***	***n***	**%**	***n***	**%**	
Total		1,331	100	683	51.31	648	48.69	
Sex								
	Boy	683	51.31					
	Girl	648	48.69					
Ethnicity								
	Han	1,287	96.69	660	96.63	627	96.76	0.90
	Minority	44	3.31	23	3.37	21	3.24	
Grade								
	7	698	52.44	352	51.54	346	53.4	0.62
	8	309	23.22	166	24.3	143	22.07	
	9	324	24.34	165	24.16	159	24.54	
Residence								
	Urban	964	72.43	478	69.99	486	75	<0.05
	Rural	367	27.57	205	30.01	162	25	
Siblings								
	None	209	15.7	149	21.82	60	9.26	<0.01
	One or more	1,122	84.3	534	78.18	588	90.74	
Perceived family affluence (mean ± sd)		3.39 ± 0.71		3.39 ± 0.74		3.38 ± 0.68		0.66
Body mass index (kg/m^2^)		18.99 ± 4.08		19.89 ± 4.53		18.04 ± 3.30		<0.01
Moderate to vigorous physical activity (days)		5.05 ± 2.08		5.43 ± 2.16		4.65 ± 1.92		<0.01
Sleep duration (hours/day)		8.42 ± 1.29		8.48 ± 1.19		8.36 ± 1.38		0.08
Screen time (hours per day)		8.60 ± 2.90		8.88 ± 2.84		8.31 ± 2.85		0.80
TV/movie time (hours/day)		2.29 ± 1.40		2.97 ± 1.43		2.86 ± 1.36		0.16
Video game time (hours/day)		2.41 ± 1.44		2.73 ± 1.50		2.21 ± 1.30		<0.01
Internet-surfing time (hours/day)		3.27 ± 1.51		3.17 ± 1.50		3.37 ± 1.51		<0.05
Anxiety (standardized scores)		40.23 ± 8.71		39.66 ± 8.16		40.82 ± 9.22		<0.05
								
Anxiety (category)								
	No	1,169	87.8	615	90	554	85.5	<0.05
	yes	162	12.2	68	10	94	14.5	
								
TV/movie time (category)								
	0–2 h	435	32.7	218	31.9	217	33.5	0.23
	2–4 h	688	51.7	351	51.4	337	52	
	4–6 h	181	13.6	95	13.9	86	13.3	
	6 h or more	27	2	19	2.8	8	1.2	
Video games time (category)								
	0–2 h	676	50.8	271	39.7	405	62.5	<0.01
	2–4 h	512	38.5	319	46.7	193	29.8	
	4–6 h	124	9.3	78	11.4	46	7.1	
	6 h or more	19	1.4	15	2.2	4	0.6	
Internet-surfing time (category)								
	0–2 h	340	25.5	182	26.6	158	24.4	0.06
	2–4 h	714	53.6	376	55.1	338	52.2	
	4–6 h	225	16.9	97	14.2	128	19.8	
	6 h or more	52	3.9	28	4.1	24	3.7	

SD, standard deviation.

The results of the association between different types of SSBs and anxiety symptoms in the overall sample and sample by sex are presented in Tables 2, 3. For overall sample, the results from GLMs indicated that only 6 or more h of video game time was positively associated with anxiety symptoms (odds ratio = 5.25, 95% CI: 1.86–14.84, *p* < 0.005). However, both TV/movie time and internet time were not associated with anxiety symptoms (both *p* > 0.05). Sex-stratified results are presented in Table 3, in boys, only time spent in video games (6 or more h) was positively associated with increased anxiety symptoms. However, in girls, different types of SSBs were not associated with anxiety symptoms (all *p* > 0.05).

**Table 2:** Association between different types of sedentary-based behaviors and anxiety symptoms in the overall sample.

**Overall**		**Odds ratio**	**95% CI**		***p***
TV/movie time					
	6 and more hours	1.76	0.62	5.04	0.290
	4–6 h	1.18	0.69	2.03	0.540
	2–4 h	1.04	0.69	1.55	0.860
	0–2 h		Reference group		
Video games time					
	6 and more hours	**5.25**	**1.86**	**14.84**	**<0.005**
	4–6 h	1.67	0.92	3.00	0.090
	2–4 h	1.42	0.97	2.08	0.075
	0–2 h		Reference group		
Internet-surfing time					
	6 and more hours	1.77	0.80	3.91	0.158
	4–6 h	1.19	0.70	2.02	0.516
	2–4 h	0.83	0.54	1.26	0.374
	0–2 h		Reference group		

TV, television; CI, confidence interval. Bold font denotes statistical significance. All the models controlled for (except the sex-stratified model, not controlling sex) variables of sex, ethnicity, grade, residence, siblings, affluence, body mass index, moderate to vigorous physical activity, and sleep duration.

**Table 3:** Association between different types of sedentary-based behaviors and anxiety symptoms in the sample by sex.

**Boys**		**Odds ratio**	**95%CI**		***p***
TV/movie time					
	6 and more hours	2.78	0.79	9.80	0.112
	4–6 h	1.61	0.71	36.31	0.256
	2–4 h	1.04	0.55	19.73	0.910
	0–2 h		Reference group		
Video games time					
	6 and more hours	**5.21**	**1.56**	**17.44**	**<0.01**
	4–6 h	1.12	0.44	28.06	0.815
	2–4 h	1.30	0.70	23.96	0.405
	0–2 h		Reference group		
Internet-surfing time					
	6 and more hours	1.39	0.41	4.65	0.598
	4–6 h	1.26	0.53	29.99	0.602
	2–4 h	0.94	0.49	18.00	0.840
	0–2 h		Reference group		
**Girls**				
TV/movie time					
	6 and more hours	0.62	0.07	5.49	0.666
	4–6 h	0.97	0.46	20.50	0.930
	2–4 h	1.02	0.60	17.42	0.938
	0–2 h		Reference group		
Video games time					
	6 and more hours	3.00	0.28	32.06	0.363
	4–6 h	2.13	0.94	48.26	0.069
	2–4 h	1.37	0.82	22.87	0.229
	0–2 h		Reference group		
Internet-surfing time					
	6 and more hours	2.02	0.67	6.07	0.212
	4–6 h	1.15	0.58	23.05	0.688
	2–4 h	0.75	0.42	13.34	0.326
	0–2 h		Reference group		

TV, television; CI, confidence interval. Bold font denotes statistical significance. All the models controlled for variables of ethnicity, grade, residence, siblings, affluence, body mass index, moderate to vigorous physical activity, and sleep duration.

## Discussion

This study sought to assess the associations between different types of SSBs and anxiety symptoms in Chinese adolescents (from Guangdong Province). The main research findings in this study were, first, that video/games time (6 or more h) was significantly and positively associated with anxiety symptoms; second, associations between different types of SSBs and anxiety symptoms varied by sex, whereas in boys, video/games time (6 or more h) was significantly and positively with anxiety symptoms; while in girls, no specific type of SSB was significantly associated with higher levels of anxiety symptoms. These research findings have notable practical implications for anxiety prevention in adolescents, especially in Chinese adolescents, which, prior to this study, were bereft of evidence.

Concordant with previous work (14, 20, 21), our study supports the assertion that excessive time spent in SSB (even one specific type) is positively associated with higher risks of anxiety in adolescents. In addition to providing supportive evidence to confirm the negative roles of SSB on mental health disorders, our study adds new evidence on the association between specific types of SSBs and anxiety symptoms, where there is little research evidence concerning the relationships between specific types of SSBs and mental health indicators in adolescents (12, 22). Indeed, the current study aimed to further discern the associations between different types of SSBs and anxiety symptoms, as well as the sex differences. Consistent with previous studies (12), video game time was significantly associated with anxiety symptoms in adolescents. Moreover, in our study, we also posit that video game time was a contributor to increased anxiety symptoms in adolescents, indicating that playing more video games is more likely to develop a mental health problem. However, our study is incongruent with some previous work, such as Mathers et al. (11), which could be attributable to methodological differences across studies, such as sample characteristics, measurement of exposure, and outcome variables, as well as covariates included in different studies. Although some prior research has attempted to unravel the underlying mechanism linking SSB and anxiety symptoms (22, 23), the mechanism linking specific types of SSBs and anxiety symptoms remains unclear (22, 23). For example, to our knowledge, it is not clear why video game time is associated with anxiety symptoms in adolescents. The possible reason may involve that contents delivered from video games negatively affect adolescents' mood (23), then generating anxiety symptoms. However, biological and neurological mechanisms explaining the association should be further clarified. Indeed, we advocate future studies explore and address this important research issue, which would be beneficial for deeper and hitherto unseen insights into the associations between SSB and anxiety in adolescents.

Studies on anxiety symptoms and TV/movie time in adolescents remain scarce (12), especially in adolescents (22, 23). However, in our study, TV/movie time and internet-surfing time were not associated with anxiety symptoms in adolescents. In line with two studies based on Canadian and Australian adolescents (12, 24), respectively, no significant associations between TV/movie and anxiety symptoms were found, which corroborates our findings. In contrast, some other studies showed that TV/movie time and internet-surfing time were significantly associated with anxiety symptoms in adolescents (12, 21, 25). These discrepant research findings may be largely owing to methodological differences. Therefore, it is advisable that research can be replicated, and confirmed or negated, in order to better understand the associations between specific types of SSBs and anxiety symptoms (as well as other mental health indicators).

Our study also found that the associations between specific types of SSBs and anxiety symptoms vary by sex. Specifically, in boys, video game time was associated with anxiety symptoms, while in girls, there were not any types of SSBs related to anxiety symptoms. To our knowledge, our study is one of the very few studies to examine the sex differences in the associations between specific types of SSBs and anxiety symptoms. Unfortunately, owing to limited and comparable evidence regarding sex differences in the associations between specific types of SSBs and anxiety symptoms, it is currently infeasible to report an overarching view (26). Despite this, some plausible explanations could be suggested for sex differences in the associations between specific types of SSBs and anxiety symptoms. The idiosyncratic preference for specific types of SSBs might be a contributing factor. For boys, they tend to have strong preferences for engaging in video games, while girls are more willing to spend time on electronic devices for social networking activities, such as internet-surfing (27). The noticeable difference might be an explanation, or at least contributory, for sex differences in the associations between specific types of SSBs and anxiety symptoms, because sex differences in preferring specific types of SSBs would make men or women to different content exposures in front of screen devices, which generates various emotional reactions. Moreover, gender-specific motivations for video games, while many girls prefer games or activities suitable for relationship maintenance, boys are often more interested in complex and competitive gameplay (28). The difference preferences could be applied to explain the sex differences in the associations between different types of SSBs and anxiety. However, more studies on the mechanism linking specific types of SSBs and anxiety for differences between sexes should be clarified in the future.

To better interpret the research findings of this study, some inherent study limitations should be mentioned. First, our study used a cross-sectional design study, which precludes causal inferences from being made. The second limitation concerns measurement in this study, a self-reported questionnaire (although being used widely across the world). This could make our measurement of SSB biased because of recall errors and other wellknown self-report limitations. Third, our study was based on samples recruited from Guangdong Province; so, our research findings may not necessarily be generalized to a broader spectrum of Chinese populations. Fourth, this study did not evaluate what time SSB occurs within a day. Knowing this information may be useful in preventing behavior-related addiction, such as SSB, from a perspective of chronobiology (29), possibly enabling mental health promotion more effectively. Fifth, we only categorized three types of SSBs (TV/movie, video games, and internet-surfing), therefore, we failed to detect more detailed information, such as the nature or content of video games (competitive or non-competitive), as well as social media use. Future studies focusing on the impact of SSB are encouraged to adopt more comprehensive measures to assess SSB. Indeed, future studies are strongly recommended to address these study limitations.

Our study has some notable practical implications, which may be significant to mental health promotion in adolescents. First, limiting SSB would likely be a beneficial approach to reduce the likelihood of anxiety in adolescents. In this regard, applying multiple strategies to reduce SSB is a feasible, and probably necessary, approach. However, among different types of SSBs, targeting specific types of SSBs for sex-tailored anxiety interventions is urgently needed, because of the significant sex differences in the associations between specific types of SSBs and anxiety symptoms in adolescents. In this regard, interventions that reduce video game time are a behavioral priority for anxiety prevention, especially for boys. In general, from the perspective of practice and policy, it is necessary to examine the efficiency of specific types of SSBs for reducing the odds of anxiety symptoms in adolescents, which could be extended to more clinically focused research.

## Conclusion

In conclusion, we found that video game time of more than 6 h per day was significantly associated with anxiety symptoms in adolescents, which can be regarded as a risk factor for poor mental health. However, for boys, video game time was associated with anxiety symptoms, while for girls, there were no associations between different types of SSBs and anxiety. Future studies are encouraged to confirm the veracity of our research findings, in an effort to discern more robust evidence. The findings of the present study could be used to inform mental health interventions with specific strategies for controlling SSB.

## Data availability statement

The original contributions presented in the study are included in the article/supplementary material, further inquiries can be directed to the corresponding author/s.

## Author contributions

SC and ZR: study design, data collection, and analyses. SC and CC: manuscript draft and edit. ZR: manuscript review and project supervision. All authors contributed to the article and approved the submitted version.

## Funding

This research was funded by Philosophy and Social Sciences in Guangdong Province, Grant Number [GD16CTY03], Humanities and Social Science, Shenzhen University, Young Teacher Award, Grant Number [17QNFC59], and Key Project of Shenzhen Education Science in 2018, Grant Number [zdfz18008].

## Conflict of interest

The authors declare that the research was conducted in the absence of any commercial or financial relationships that could be construed as a potential conflict of interest.

## Publisher's note

All claims expressed in this article are solely those of the authors and do not necessarily represent those of their affiliated organizations, or those of the publisher, the editors and the reviewers. Any product that may be evaluated in this article, or claim that may be made by its manufacturer, is not guaranteed or endorsed by the publisher.
